# Dependence on MUC1-C in Progression of Neuroendocrine Prostate Cancer

**DOI:** 10.3390/ijms24043719

**Published:** 2023-02-13

**Authors:** Donald Kufe

**Affiliations:** Dana-Farber Cancer Institute, Harvard Medical School, Boston, MA 02215, USA; donald_kufe@dfci.harvard.edu

**Keywords:** MUC1-C, NEPC, CSC, lineage plasticity, chromatin remodeling

## Abstract

Castration resistant prostate cancer (CRPC) is responsive to androgen receptor (AR) axis targeted agents; however, patients invariably relapse with resistant disease that often progresses to neuroendocrine prostate cancer (NEPC). Treatment-related NEPC (t-NEPC) is highly aggressive with limited therapeutic options and poor survival outcomes. The molecular basis for NEPC progression remains incompletely understood. The *MUC1* gene evolved in mammals to protect barrier tissues from loss of homeostasis. *MUC1* encodes the transmembrane MUC1-C subunit, which is activated by inflammation and contributes to wound repair. However, chronic activation of MUC1-C contributes to lineage plasticity and carcinogenesis. Studies in human NEPC cell models have demonstrated that MUC1-C suppresses the AR axis and induces the Yamanaka OSKM pluripotency factors. MUC1-C interacts directly with MYC and activates the expression of the BRN2 neural transcription factor (TF) and other effectors, such as ASCL1, of the NE phenotype. MUC1-C also induces the NOTCH1 stemness TF in promoting the NEPC cancer stem cell (CSC) state. These MUC1-C-driven pathways are coupled with activation of the SWI/SNF embryonic stem BAF (esBAF) and polybromo-BAF (PBAF) chromatin remodeling complexes and global changes in chromatin architecture. The effects of MUC1-C on chromatin accessibility integrate the CSC state with the control of redox balance and induction of self-renewal capacity. Importantly, targeting MUC1-C inhibits NEPC self-renewal, tumorigenicity and therapeutic resistance. This dependence on MUC1-C extends to other NE carcinomas, such as SCLC and MCC, and identify MUC1-C as a target for the treatment of these aggressive malignancies with the anti-MUC1 agents now under clinical and preclinical development.

## 1. Background

Castration resistant prostate cancer (CRPC) is effectively treated with agents, such as enzalutamide and abiraterone, that target the androgen receptor (AR) axis [1]. However, patients with CRPC invariably develop resistance to AR pathway-targeted therapy and often progress to a more aggressive form with neuroendocrine (NE) features [2,3,4,5]. As a result, the incidence of treatment associated neuroendocrine prostate cancer (t-NEPC) has been increasing with the now widespread use of AR-targeted agents [4,5]. Poorly differentiated NE carcinoma of the prostate can also develop de novo with pathologies similar to that of small-cell lung cancer (SCLC) and other types of small cell carcinomas [6,7]. Despite treatment with docetaxel, cabazitaxel and platinum-based chemotherapy, the median overall survival (OS) of patients with t-NEPC and de novo NEPC has remained poor. Immune checkpoint inhibitors have also had limited success in the treatment of CRPC and NEPC [8,9,10]. In this regard, patients who progress to t-NEPC have a median OS of <1 year, whereas those with de novo NEPC have a median OS of 16.8 months from diagnosis [4,5,11]. Notably, there are presently few effective targeted agents for NEPC treatment.

Resistance to AR pathway-targeted agents is associated with significant increases in truncated AR splice variant-7 (AR-V7) expression [12,13,14]. Anti-AR-V7 agents have been under development, although they have not been approved as yet for advanced CRPC or NEPC therapy [15]. DNA damage repair pathways are dysregulated in metastatic PC and therefore represent potential vulnerabilities for treatment [16,17]. Along these lines, PARP inhibitors, such as olaparib, have extended relapse-free survival of patients with CRPCs that harbor mutations in homologous recombination repair (HRR) genes [18]. Based on these findings, olaparib was approved for the treatment of enzalutamide or abiraterone unresponsive mCRPCs with certain HRR gene mutations [19]. Trials targeting PI3K/AKT [20], Aurora kinase A [21] and MEK [22] are underway based on their dysregulation in advanced CRPC and in progression to NEPC. In addition, RET has been identified as a target based on the overexpression of this tyrosine kinase in NEPC [23].

As another potential target, the *MUC1* gene is aberrantly expressed in advanced CRPC and NEPC [24]. *MUC1* is amplified in 30% of a CRPC cohort with NEPC enrichment compared to 6% in the SU2C CRPC cohort and 2% in the TCGA primary PC cohort [24]. In addition, MUC1 expression is significantly increased in CRPCs compared to localized, hormone-naïve PCs [24]. Upregulation of MUC1 in advanced CRPC is associated with (i) Gleason grades ≥ 7, aggressive disease and increased risk of recurrence [25,26,27], (ii) early biochemical failure and PC-related death [28,29], and (iii) bone metastases [30]. These findings supported the potential of MUC1 as a target for advanced CRPC and NEPC treatment.

### 1.1. Evolution of the MUC1 Gene for the Adaptation of Barrier Tissues

*MUC1* was discovered based on the overexpression of this gene in human breast cancers [31,32]. The cloning of the *MUC1* gene identified a unique structure with variable numbers of conserved 60 base-pair tandem repeats (TRs) [33,34]. Further characterization demonstrated frequent alterations of the *MUC1* gene in breast cancers [35]. Of interest were the subsequent findings that *MUC1* first appeared in mammals, which supported a fundamental role in placentation and lactation [36,37].

**Figure 1:**
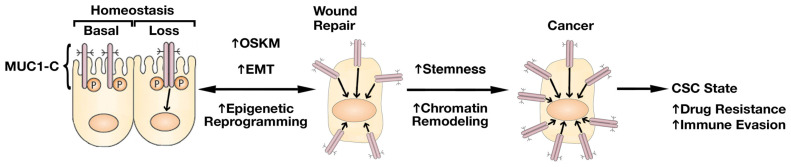
**The activation of MUC1-C by loss of homeostasis contributes to wound healing and progression to cancer.** The transmembrane MUC1-C subunit is expressed at the apical borders of polarized epithelial cells where it is poised to respond to stress. The activation of MUC1-C in response to loss of homeostasis induces the Yamanaka pluripotency factors, EMT and epigenetic reprogramming. MUC1-C also contributes to inflammatory, proliferative and remodeling responses associated with wound repair. These responses are, in principle, reversible with healing; however, prolonged activation of MUC1-C in settings of chronic inflammation with the remodeling of chromatin drive progression to cancer. Figure modified from [43].

*MUC1* encodes a polypeptide that undergoes autocleavage into N-terminal (MUC1-N) and C-terminal (MUC1-C) subunits [38]. In turn, MUC1-N and MUC1-C form a noncovalent complex which localizes to the cell membrane [38]. MUC1-N contains 20 aa TRs that are abundantly modified by *O*-glycosylation and extends from the cell surface into a protective mucous barrier [38]. MUC1-C is the transmembrane component of the heterodimer, containing a 58 aa extracellular domain, 28 aa transmembrane region and a 72 aa cytoplasmic tail [38]. The MUC1-N/MUC1-C complex plays a role in physically protecting barrier tissues from infections [39]. This barrier function also plays a role in embryo implantation and placentation [40,41]. Importantly, further evidence has indicated that MUC1-C evolved in mammals to confer the adaptation of barrier tissues, including resident stem cells and immune cells, to loss of homeostasis (Figure 1) [42].

The activation of MUC1-C in the response of barrier tissues to stress induces inflammatory, proliferative and remodeling pathways that are associated with wound healing and repair [42,44]. MUC1-C represses CRB3 and E-cadherin expression in promoting loss of polarity [44]. MUC1-C also induces (i) the epithelial-mesenchymal transition (EMT) by activating the expression of the ZEB1, TWIST1 and SNAIL EMT TFs, and (ii) epigenetic reprogramming by the activation of Polycomb Repressive Complex 1 (PRC1) and PRC2 (Figure 1) [44,45]. These responses to loss of homeostasis are conceptually reversible with wound repair; however, prolonged MUC1-C activation in response to chronic inflammation imprints these changes with progression to cancer (Figure 1) [42,43,44]. As a consequence, MUC1-C has the capacity to promote carcinogenesis in barrier tissues, such as the prostatic epithelium, that are subject to chronic inflammatory responses [42]. Moreover, chronic inflammation associated with diet, injury and an altered microbiome has been implicated in driving prostate carcinogenesis [46]. Prostatitis is associated with EMT [47,48] and prostate cancer [49], indicating that the prolonged activation of MUC1-C in settings of chronic prostatitis could also contribute to PC progression.

### 1.2. Importance of MUC1-C in Suppressing the AR Axis and Driving CRPC→NEPC Progression

MUC1-C drives lineage plasticity and progression to the CSC state in human cancers [42,44]. Studies in AR-dependent LNCaP cells selected for proliferation in androgen-depleted medium demonstrated upregulation of MUC1-C expression [24]. The androgen-independent LNCaP-AI cells also exhibited downregulation of AR axis signaling [24], in concert with the finding that MUC1-C suppresses AR expression in PC cells [50]. In further support for the repression of the AR axis, silencing MUC1-C in this model activated PSA/KLK3, NKX3.1 and TMPRSS2 expression [24]. This inverse relationship between MUC1 and AR signaling was extended by the observation that MUC1-high CRPC tumors associate with decreased AR, KLK3, TMPRSS2, HERC3 and NKX3-1 expression levels.

**Figure 2:**
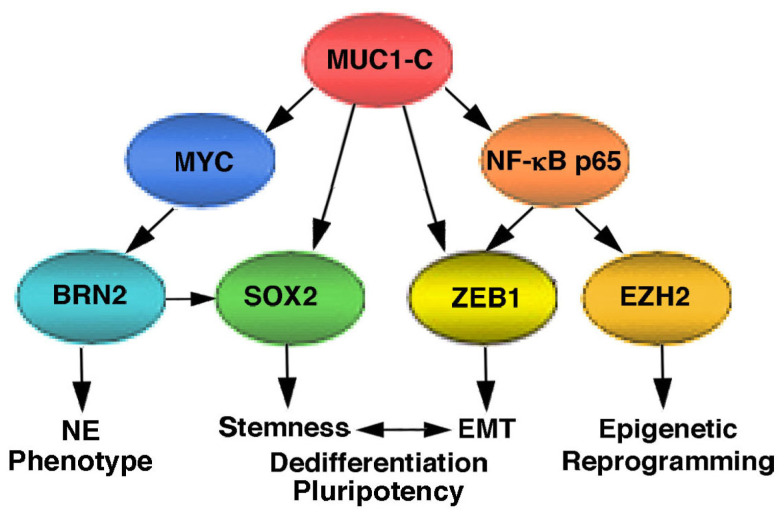
**MUC1-C drives NEPC dedifferentiation.** MUC1-C binds directly to the MYC HLH/LZ domain and contributes to the induction of MYC target genes. MUC1-C/MYC complexes occupy the BRN2 promoter and induce BRN2 expression. BRN2 induces SOX2 expression [51]. In addition, MUC1-C drives KLF4 and OCT4, which are collectively referred to as OSKM factors, and are sufficient for inducing pluripotency and dedifferentiation of somatic cells [57]. In addition, MUC1-C activates the inflammatory NF-κB p65 pathway and, by binding directly to NF-κB p65, promotes the activation of NF-κB p65 target genes [61], including (i) ZEB1 and thereby EMT and stemness, and (ii) EZH2 and epigenetic reprogramming [45,62]. In this way, MUC1-C integrates activation of the MYC and NF-κB p65 pathways to drive NEPC dedifferentiation and self-renewal. Figure modified from [24].

BRN2 is a neural transcription factor that drives SOX2, induces NE markers and enriches for an NEPC gene signature [51]. DU-145 PC cells, derived from a brain metastasis [52], express BRN2 and other genes associated with the NE phenotype [51,53]. Studies in LNCaP-AI, DU-145 and H660 NEPC [21] cells uncovered a common role for MUC1-C in activating BRN2 expression in association with upregulation of MYC, SOX2 and the NE phenotype (Figure 2) [24]. The finding that MUC1 expression in CRPC tumors associates with upregulation of BRN2, SOX2 and the NEPC score provided additional evidence that, in parallel with the suppression of the AR axis, MUC1-C promotes CRPC→NEPC progression [24]. In further support for the notion that MUC1-C drives NEPC dedifferentiation, MUC1-C was necessary for the induction of MYCN, EZH2 and the ASCL1, AURKA and SYP NE markers, which have been associated with progression to NEPC (Figure 2) [21,54]. NE dedifferentiation vs. transdifferentiation is used here in that MUC1-C induces the NE phenotype in association with stemness, as evidenced by the induction of self-renewal capacity and tumorigenicity [24].

t-NEPC is associated with the activation of gene programs that drive lineage plasticity, the CSC state and NE dedifferentiation [2,3,4,55,56]. The observation that MUC1-C induces MYC and SOX2 was extended to include KLF4 and OCT4, which collectively represent the Yamanaka OSKM pluripotency factors that are sufficient to confer lineage plasticity and dedifferentiation of fibroblasts [57]. Pluripotency factors are repressed in somatic cells to maintain lineage specification, whereas they are induced transiently in wound healing and persistently in cancer [58]. Lineage plasticity and stemness in cancer contributes to progression and treatment resistance [3,59,60]. In line with promoting lineage infidelity, the upregulation of MUC1-C in NEPC progression supported the notion that MUC1-C is also necessary for the NEPC CSC state. Indeed, targeting MUC1-C genetically and pharmacologically suppressed NEPC cell self-renewal and tumorigenicity [24].

### 1.3. MUC1-C Drives NEPC Cell Stemness by Activating the SWI/SNF BAF Chromatin Remodeling Complex

The seminal findings that NEPC CSCs are dependent on MUC1-C for self-renewal capacity uncovered a new line of investigation; that is, more precisely determining how MUC1-C drives the NEPC CSC state. Certain insights in this regard emerged from work on the involvement of MUC1-C in activating the PRC1/2 complexes in cancer cells [43,62,63]. The mammalian SWI/SNF BRG/BRAHMA factor (BAF) chromatin remodeling complex intersects with modifications of nucleosomes by PRC1/2 in regulating gene expression and cell fate [64,65,66,67]. Canonical core BAF subunits include (i) a BRG1/SMARCA4 or BRM/SMARCA2 ATPase, (ii) SMARCB1, which is essential for targeting enhancers, (iii) ARID1A/B, which maintain BAF on enhancers, and (iv) SMARCC1/2 leucine zipper proteins [68,69,70,71]. The ETS family transcription factor ERG requires BAF for mediating prostate oncogenesis [72], whereas little had been known about the regulation of BAF in PC cells.

**Figure 3:**
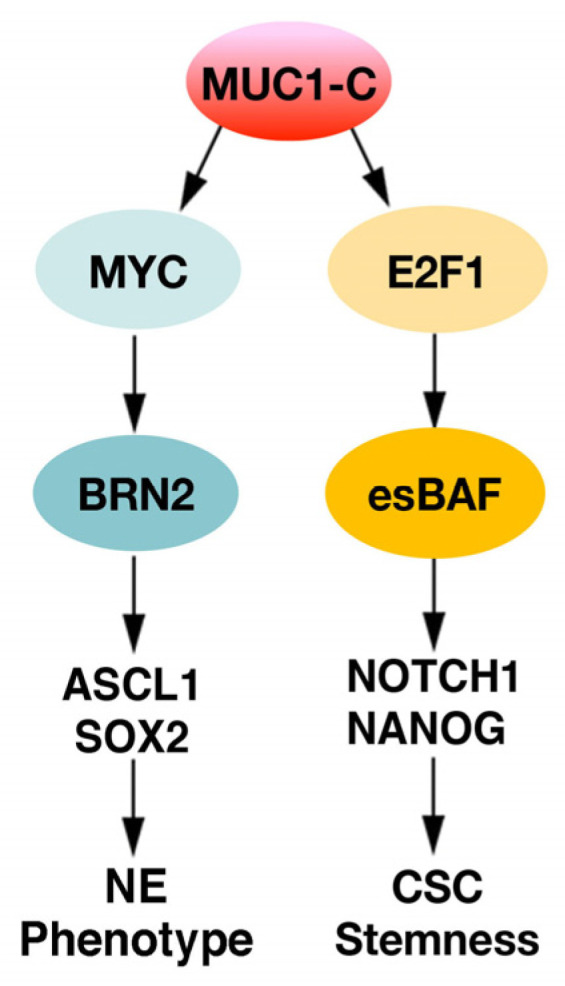
**MUC1-C activates the esBAF chromatin remodeling complex in driving the NEPC CSC state.** In parallel with the induction of the BRN2 and NE phenotype, MUC1-C interacts with E2F1 and induces the expression of the esBAF subunits. MUC1-C forms a nuclear complex with BRG1 and ARID1A, which activates the NOTCH1 gene, NOTCH1 expression and NOTCH1 target genes. MUC1-C-induced activation of esBAF also promotes NANOG expression and self-renewal capacity. These findings demonstrate that MUC1-C integrates the MYC and E2F1 pathways in driving NEPC dedifferentiation. Figure modified from [73].

The finding that MUC1-C activates BAF in NEPC cells thus opened a new line of investigation for the involvement of this SWI/SNF complex in NEPC progression [73]. The binding of MUC1-C to the E2F1 TF in NEPC cells was found to induce the expression of BRG1 and ARID1A, as well as SMARCD1/BAF60a, SMARCC1/BAF155 and SMARCC2/BAF170 [73], which are components of the embryonic stem cell (ESC)-specific BAF (esBAF) complex [74,75]. esBAF is required for regulating ESC gene expression and thereby ESC self-renewal and differentiation [76]. Notably in this regard, MUC1-C associates with ESC and CSC gene signatures in NEPC cells [73]. From these results, *NOTCH1*, which has been linked to stemness and driving CRPC [77], was identified as a MUC1-C-activated gene that is also dependent on BRG1 and ARID1A for expression (Figure 3) [73]. In addition, the MUC1-C→esBAF pathway was found to induce the NANOG pluripotency factor which promotes stemness (Figure 3) [78]. Consistent with these findings, MUC1-C→esBAF signaling was necessary for NEPC cell self-renewal capacity [73]. These findings further highlighted the involvement of MUC1-C in activating (i) MYC with the induction of pluripotency and the NE phenotype [24], and (ii) E2F1→esBAF in inducing NOTCH1, NANOG and the CSC state (Figure 3) [73].

### 1.4. MUC1-C Activates the SWI/SNF PBAF Chromatin Remodeling Complex in Integrating Redox Balance with Stemness of NEPC Cells

**Figure 4:**
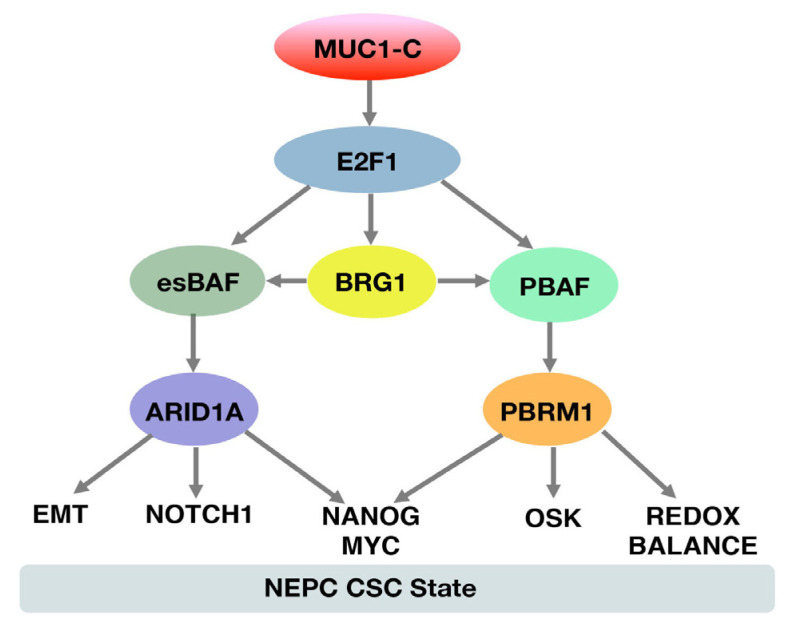
**MUC1-C→E2F1 signaling integrates the activation of the esBAF and PBAF chromatin remodeling complexes.** MUC1-C forms nuclear complexes with E2F1 that activate the expression of the esBAF and PBAF subunits. MUC1-C associates with PBRM1 and NRF2 in increasing the chromatin accessibility of NRF2 target genes, including SLC7A11, G6PD and PGD, that regulate redox balance. MUC1-C-induced activation of PBRM1/PBAF also contributes to the expression of the OSKM + NANOG pluripotency factors and integration with the ARID1A/esBAF complex that drives EMT, NOTCH1 and the NEPC CSC state. Figure modified from [84].

The polybromo-associated BAF (PBAF) chromatin remodeling complex includes the BRG1 ATPase, PBRM1/BAF180, ARID2/BAF200 and BRD7 [64]. BRG1 is shared with BAF and PBAF [64], whereas PBRM1 has a distinguishing capacity in PBAF with functions in (i) DNA damage-associated transcriptional repression and DNA repair [79,80], and (ii) regulation of genes involved in the oxidative stress response and apoptosis [81]. In addition, ARID2 regulates IFN-induced genes [82] and BRD7 interacts with BRCA1-mediated transcription [83]. Interestingly, as found for esBAF [73], the MUC1-C→E2F1 pathway was essential for the induction of PBRM1, ARID2 and BRD7 expression in NEPC cells (Figure 4) [84]. As also reported for esBAF (BRG1, ARID1A), MUC1-C/E2F1 complexes were detectable on the *PBRM1*, *ARID2* and *BRD7* promoter regions containing E2F binding motifs [84]. These findings, and those demonstrating that MUC1-C→E2F1 signaling activates genes encoding PRC2 (EZH2, SUZ12 and EED) subunits, indicated that MUC1-C plays a role in integrating functions of the PRC2 and SWI/SNF BAF and PBAF complexes in NEPC cells [45,62,73,84].

The MUC1-C→esBAF pathway contributes to the NEPC CSC state [73]. CSCs are effective in repairing DNA damage and controlling ROS levels [85,86]. In support of MUC1-C in maintaining redox balance in CRPC CSCs, MUC1-C-induced PBRM1 expression associated with the activation of NRF2 target genes (Figure 4) [73]. The NRF2 TF is a master regulator of the anti-oxidant response and driver of cancer progression and resistance to therapy [87,88]. MUC1-C formed a complex with NRF2 and PBRM1 on the *SLC7A11* gene [84], which encodes the xCT cystine-glutamate antiporter, a subunit of the Xc- system that confers cysteine uptake for GSH synthesis [89]. In this way, MUC1-C, NRF2 and PBRM1 were necessary for increasing the chromatin accessibility of the *SLC7A11* gene and for xCT expression [84]. MUC1-C/NRF2/PBRM1 complexes also contributed to the chromatin accessibility of the *G6PD* gene and expression of glucose-6-phosphate dehydrogenase (G6PD), which converts NADP+ to NADPH [84]. In accordance with this MUC1-C-driven activation of *SLC7A11*, *G6PD* and other antioxidant genes, silencing MUC1-C and PBRM1 in NEPC cells decreased GSH, GSH/GSSG and NADP/NADPH levels in association with increases in sensitivity to oxidative stress [84]. Of further interest, MUC1-C functioned in cross-talk between the esBAF and PBAF complexes in integrating the expression of pluripotency and stemness factors with effectors of redox balance (Figure 4) [84]. These findings for MUC1-C-induced regulation of esBAF and PBAF formed the basis for studies of MUC1-C involvement in the remodeling of chromatin in NEPC CSCs.

### 1.5. MUC1-C Regulates Chromatin Accessibility across the Genome of NEPC Cells in Promoting the CSC State

Chromatin remodeling is critical for lineage plasticity, EMT and the CSC state [90]. MUC1-C drives the esBAF and PBAF chromatin remodeling complexes in NEPC CSCs [73,84]. By extension, ATAC-seq studies showed that MUC1-C is associated with global changes in chromatin accessibility across the genome of DU-145 cells [90]. Silencing MUC1-C identified Differentially Accessible Regions (DARs) with increases and decreases in chromatin accessibility [90]. DARs were located at proximal regions within 1–3 kb and distal intergenic regions within 50–500 kb from annotated TSSs [90]. Associations of MUC1-C-induced DARs with Differentially Expressed Genes (DEGs) identified genes enriched for FOS, JUN and NEF2 binding motifs that are recognized by members of the AP-1 family of TFs. *NOTCH1* was identified among these genes in concert with the demonstration that MUC1-C induces NOTCH1 in driving the self-renewal of NEPC CSCs [73,90]. In further support of esBAF involvement in activating *NOTCH1* [73], MUC1-C occupied a *NOTCH1* proximal enhancer-like sequence (pELS) with JUN and ARID1A in association with increases in chromatin accessibility, H3K4 trimethylation and NOTCH1 expression [90]. Similar results were obtained for the *EGR1* stemness gene, indicating that MUC1-C activates a JUN- and esBAF-dependent pathway of importance for increasing the chromatin accessibility of genes that promote the NEPC state [90]. These findings indicated that MUC1-C drives NEPC progression by inducing JUN-mediated chromatin remodeling, which parenthetically is essential for the wound healing response and maintaining tissue homeostasis [42,91].

**Figure 5:**
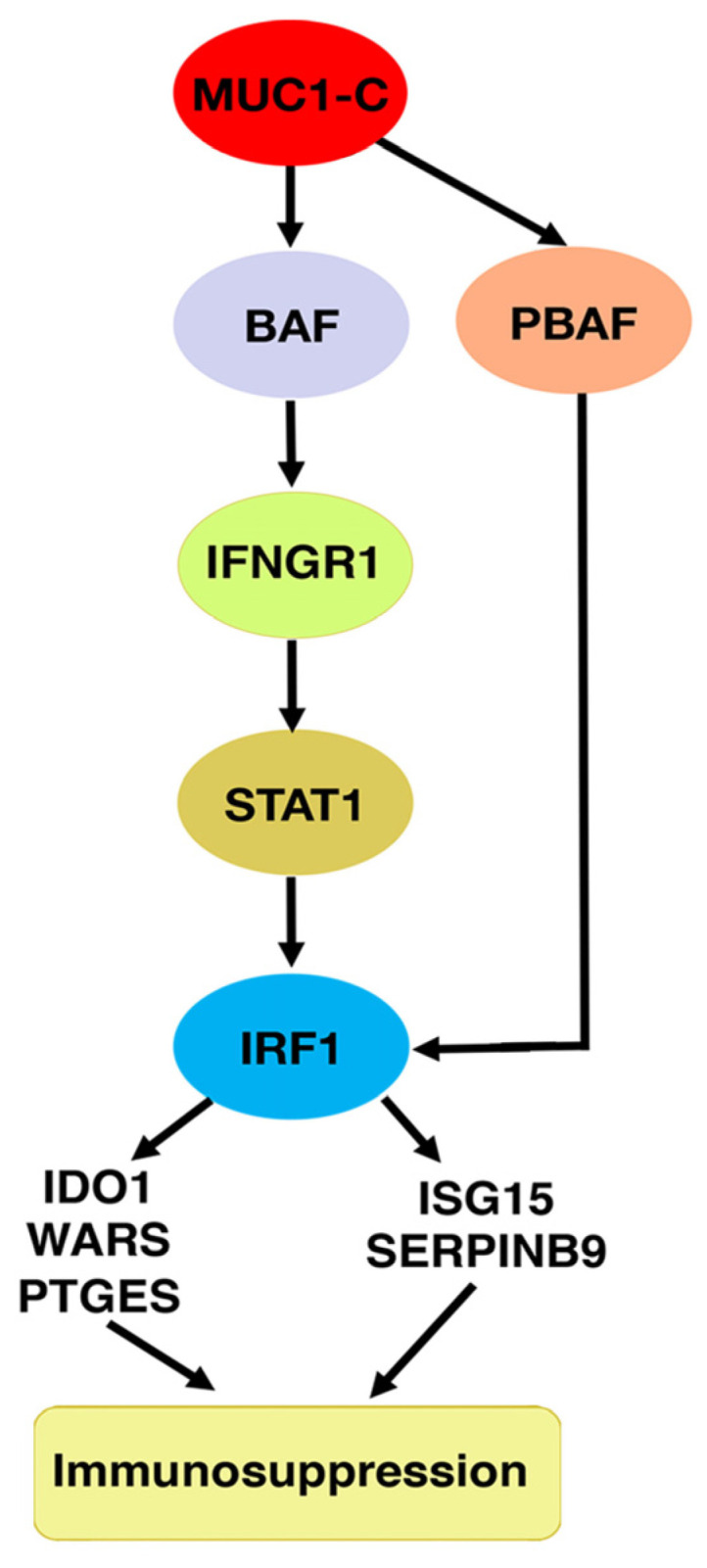
MUC1-C integrates the induction of the esBAF and PBAF chromatin remodeling complexes with the chronic activation of the IFNG pathway and immunosuppression. MUC1-C activates the IFNGR1 gene by forming a complex with JUN and ARID1A that increases chromatin accessibility, H3K4 trimethylation and IFNGR1 expression. MUC1-C thereby contributes to upregulation of STAT1 and IRF1, and in turn interacts with IRF1 and PBRM1 to drive the expression of (i) IDO1, WARS and PTGES that metabolically suppress the TME, and (ii) ISG15 and SERPINB9, which inhibit T cell function. Consistent with the induction of these immunosuppressive effectors, MUC1 associates with immune cell-depleted cold TMEs. Figure modified from [92].

The distinction between MUC1-C-induced activation of the esBAF and PBAF complexes in driving NEPC progression was further investigated with studies of their potential involvement in inflammatory signaling pathways (Figure 5). Along this line of investigation, MUC1-C contributes to the inflammatory wound healing response and prolonged activation of MUC1-C by chronic inflammation promotes oncogenesis [42,44]. An analysis of the TCGA-PRAD and SU2C-CRPC datasets demonstrated that MUC1-high PCs significantly associate with the activation of the type II IFN response gene signature [92]. Moreover, MUC1-high PCs had significantly increased levels of IFNGR1, STAT1 and IRF1 [92], which drive IFN response genes (ISGs) and chronic inflammation in cancer cells [93]. MUC1-C was found to be necessary for IFNGR1, STAT1 and IRF1 expression in NEPC cells (Figure 5) [92]. In further support for the interactions among MUC1-C, JUN and esBAF [90], MUC1-C/JUN/ARID1A complexes were detectable on the *IFNGR1* gene at a distal enhancer-like signature (dELS) in association with increases in chromatin accessibility, H3K4me3 levels and IFNGR1 expression [92]. The stimulation of the IFNGR1 complex by IFN-gamma (IFNG) activates the STAT1→IRF1 pathway [93]. Silencing MUC1-C decreased the chromatin accessibility of the *STAT1* and *IRF1* genes and their expression (Figure 5) [92]. In addition, MUC1-C and PBRM1 were necessary for the expression of the downstream immunosuppressive effectors (i) indoleamine-2,3-dioxygenase (IDO1) [94], (ii) tryptophanyl-tRNA synthetase (WARS) [95], and (iii) prostaglandin E synthase (PTGES) [96] (Figure 5). In support of these results in NEPC cells, MUC1-high PC tumors significantly associate with upregulation of IDO1, WARS and PTGES expression [92]. Of additional importance, MUC1-high CRPC tumors associate with the suppression of the TME, as evidenced by (i) negative regulation T cell and NK cell mediated immunity, and (ii) decreases in CD4+ memory T cells, Th2 cells, M2 macrophages and the ImmuneScore [92]. An analysis of a scRNA-seq dataset further demonstrated that MUC1 associates with CSC and IFN signatures across individual CRPC cells [92].

### 1.6. Involvement of MUC1-C in Lineage Plasticity of NE Cancers

The lineage plasticity of the NE phenotype is of importance to the progression and response of prostate and other types of cancers [97]. An analysis of CRPC, NEPC and small cell lung cancer (SCLC) tumors identified pan-cancer convergence to a small cell NE phenotype, characterized by *TP53* and *RB1* loss, common epigenetic alterations and the expression of NE markers [98]. The findings that MUC1-C drives lineage plasticity in the progression to NEPC suggested that MUC1-C may play a role in other cancers with NE dedifferentiation or transdifferentiation. In support of that notion, MUC1-C dictates NE lineage specification in pancreatic ductal carcinomas (PDAC-NE) (Table 1) [99]. As found in NEPC [24,92], MUC1-C induces the Yamanaka OSKM pluripotency factors, inflammatory IFN signaling and the NOTCH1/2 TFs in PDAC-NE cells [99]. In addition, MUC1-C interacted with MYC in activating the BRN2 and ASCL1 neural TFs and thereby the expression of NE markers [99]. In SCLC, MUC1-C activates the MYC pathway in classic NE SCLC-ASCL1 (SCLC-A), variant NE SCLC-NEUROD1 (SCLC-N) and non-NE SCLC-POU2F3 (SCLC-P) subtypes (Table 1) [100]. MUC1-C→MYC signaling was necessary for the induction of (i) NOTCH2, which is a marker of pulmonary NE stem cells, and (ii) the ASCL1 and NEUROD1 neural TFs [100]. Merkel cell carcinoma (MCC) is another aggressive NE malignancy [101]. MUC1 is dysregulated in Merkel cell polyomavirus (MCPyV)-positive and -negative MCCs [101]. In both MCC subtypes, MUC1-C interacts with MYCL in inducing the expression of the OSKM + NANOG pluripotency factors and the neural BRN2, NEUROD1 and ATOH1 TFs (Table 1) [101].

These studies have identified common MUC1-C-driven pathways in NE cancers that include the activation of (i) MYC family members, (ii) Yamanaka pluripotency factors, (iii) NE lineage dictating TFs and (iv) NOTCH stemness TFs. Moreover, targeting MUC1-C genetically and pharmacologically in NEPC, PDAC-NE, SCLC and MCC cells inhibits tumorsphere formation and tumorigenicity (Table 1) [24,92,99,100,101], in support of their dependency on MUC1-C for self-renewal. These findings have identified MUC1-C as a potential target for the treatment of NEPC and other recalcitrant NE cancers that have limited therapeutic options. To that end, the generation of MAb 3D1 against the MUC1-C extracellular domain provided an opportunity for the development of agents that target MUC1-C on the cancer cell surface [102]. As one example, an allogeneic anti-MUC1-C CAR T cell using MAb 3D1 sequences is undergoing Phase I evaluation for the treatment of MUC1-C-expressing cancers (NCT05239143: P-MUC1C-ALLO1 Allogeneic CAR-T Cells in the Treatment of Subjects with Advanced or Metastatic Solid Tumors). In addition, anti-MUC1-C huMAb3D1-MMAE ADCs are under development by the NCI NExT Program for IND-enabling studies and performing early phase clinical trials in recalcitrant cancers.

## 2. Conclusions

In summary, the discoveries described here indicate that MUC1-C contributes to NEPC progression by activating pathways that drive lineage infidelity, epigenetic reprogramming and chromatin remodeling [24,73,84,91,92]. In this way, MUC1-C promotes the NEPC CSC state, DNA damage resistance and immune evasion. These findings highlight MUC1-C as a target for the treatment of NEPC and certain other aggressive NE cancers with the anti-MUC1-C agents that are now under development.

## Figures and Tables

**Table 1:** Common MUC1-C Dependencies in NE Carcinomas.

Dependency	NEPC	PDAC-NE	SCLC	MCC
**MYC Family**	MYC	MYC	MYC	MYCL
**Pluripotency Factors**	OSKM + NANOG	OSKM	ND *	OSKM + NANOG
**Neural TFs**	BRN2 ASCL1	BRN2 ASCL1 NEUROD1	BRN2 ASCL1	BRN2 NEUROD1 ATOH1
**Stemness Factors**	NOTCH1 BMI1	NOTCH1 NOTCH2	NOTCH1 BMI1	BMI1
**Tumorsphere** **Formation**	+	+	+	+
**Tumorigenicity**	+	+	+	+

* ND: Not detected.

## Data Availability

Not applicable.
